# Letter from Paris

**Published:** 1868-02

**Authors:** 


### LETTER FROM PARIS.

PARIS, *November 24th, 1867*.

The *Bulletin de Therapeutique* has contained in its three last numbers an extremely judicious essay on

DELIVERY OF THE AFTER-BIRTH

in cases of miscarriage, of which it is worth while to give you an abstract. Dr. Gueniot, the author, observes that among other differences between a miscarriage and a regular labor, it is noticeable that in the former, the expulsion of the fœtus and detachment of the placenta begin to take place at the same time, instead of constituting two orders of phenomena essentially distinct, as in the latter case. Hence in abortion, hemorrhage is associated with the uterine contractions, throughout their entire duration.

On the other hand, the *complete* separation of the placenta is tardy of accomplishment, since the adherences are relatively more solid during the first months of gestation, and the contracting power of the uterus little developed. Moreover, even when the placenta be entirely detached from the uterus, the length and firmness of the neck of the womb opposes unusual obstacles to the expulsion of the after-birth. In view of all these considerations, it is easy to see that the danger of miscarriage belongs, not to the movement of the expulsion of the fœtus, which is usually unaccompanied by any difficulty, but to the separation and expulsion of the placenta. During this period, the patient is exposed to accidents resulting either from hemorrhage, or from retention and decomposition of the placental mass. Putrid infection, metritis, anemia, nervous exhaustion, shiverings, fever vomiting, dysuria, and various neuralgias may be expected in this connection. Finally, uterine phlebitis and metastatic abscesses may complicate a miscarriage, as in an unfortunate patient observed by Dr. Gueniot at the maternité.

The therapeutic problem suggested by the consideration of these possibilities, consists in determining the line of conduct required in different cases of miscarriages, to favor the complete repulsion of the placenta and its membranes, and to combat the various accidents which may have been determined by their retention. The necessities of the case vary greatly, according to the period of gestation, at which the miscarriage has taken place. During the first two months, if the placenta be not expelled together with the embryon, its spontaneous delivery is extremely slow, and the process may be prolonged two days, without entailing any danger upon the patient. Until, therefore, the period has elapsed, the physician is not called upon to interfere, unless in case of accident.

During the third and fourth month, the membranes are ordinarily ruptured at any early stage of the labor, before the placenta be completely detached, or the os sufficiently dilated. On this account, the placenta is expelled after the fœtus; but the limit of safety in the delay must be shortened to twenty-four hours. Finally, in the fifth and sixth months, so much greater facilities exist for the expulsion of the after-birth, that its retention must be considered abnormal if it last more than twelve hours for the fifth, or six for the sixth month.

The precision of these limits is of the utmost importance, as then it affords the physician the first indications for deciding whether to intervene with the resources of art, or permit nature to take its course.

The author then classifies abortion into five types:

1st. The abortion has taken place, the delivery of the placenta has not, or it is incomplete, and no accident has occurred.

2d. Same situation as the above, but complicated by accidents.

3d. Abortion is complete, but the physician is in doubt whether the placenta be expelled or not.

4th. Fœtus and placenta had both been expelled, but accidents have occurred claiming intervention.

5th. Finally, abortion has not yet occurred, but it is recognized to be inevitable.

In the first case, as soon as the time fixed for the normal expulsion of the placenta has been overpassed, the physician has reason to *certainly* anticipate accidents, even if they have not already occurred. Moreover, the divers methods of intervention will become difficult of employment with every moment of delay, consequently, the indications for prompt interference are sufficiently pressing. On the other hand, this interference is always accompanied by certain dangers, and the decision for action or inaction, must in each case be based on a special comparison between present inconveniencies and future perils. It is certain, in all cases, that immunity from accident during several days retention of the placenta can only be observed when it has remained nearly completely adherent; as soon as it begins to separate, it is liable to putrefy.

The danger varies, moreover, according to the different periods already described. In the first, the small development of the uterine vessels saves the patient from the chance of dangerous hemorrhage, and the small size of the placenta renders its putrefaction a matter of less consequence. Hence chills, fevers, a certain pallor of the face, diarrhœa, and dryness of the tongue, are generally the greatest accidents to which the patient is exposed. Consequently, in view of the various inconveniences of active intervention, it is better for the physician to restrain his efforts to vigilant surveillance, in all cases of retention of the placenta after abortion, occurring in the two first months of gestation.

During the second period of gestation, the hemorrhage, though much more abundant, can still be readily mastered, by means of the vaginal tampon. But, after expulsion of the fœtus, the os contracts promptly, and speedily opposes a serious obstacle to the natural or artificial delivery of the placenta. A considerable mass is retained to putrefy, and may occasion a mortal infection. Hence the indication is urgent to deliver the woman artificially, as soon as possible, after twenty-four hours have elapsed without spontaneous delivery.

M. Gueniot then reviews the various agents proposed for effecting this delivery. He rejects traction on the umbilical cord, on account of its fragility. He admits that the index finger may occasionally be employed, but it is necessary the uterine neck be sufficiently open to give free passage to the finger, that the vagina permit the entrance of the hand, to the close vicinity of the os; finally, that it be possible to separate the placenta *en masse.*

The first condition is extremely rare a few hours after abortion, and never exists after several days. The realization of the second occasions great pain, and exposes the patient to contusions and lacerations. Finally, the third, the most important of all, is necessarily problematic. Hence the finger is to be regarded as a precarious resource. Pincers, curettes, hooks, are all more or less dangerous. Intra-uterine injections can only be employed with a return-current, which greatly diminishes their efficacy.

Having thus reviewed these various agents, M. Gueniot excludes them all in favor of three, upon which he places immense reliance, the prepared sponge, the uterine dilator, and the ergot of rye.

The dilator, you know, consists of a small India-rubber bag, introduced into the uterine cavity in a collapsed form, and then filled out with warm water. This, in virtue of a continued irritation produced on the internal sphincter, provokes uterine contractions nearly inevitably at the end of several hours. The bag constitutes an artificial substitute for the natural membranes that have been prematurely ruptured, and its action imitates nature as closely as possible. Not only provokes uterine contractions, but it mechanically enlarges the os, and, moreover, performs the office of a tampon in moderating the hemorrhage. For this last purpose, however, the caoutchouc bag is no more infallible than the embryonic mass itself. M. Gueniot considers the dilator one of the most precious means for provoking the delivery of the placenta.

Where the prepared sponge is inserted in the uterine neck, it is recommended to accompany it by a vaginal tampon, as better security against hemorrhage.

Finally, ergot may be administered in doses of two grammes, (thirty-two grains,) and on M. Gueniot's testimony, is certain to determine uterine contractions in from twelve to eighteen hours. If the action is not sufficiently marked after the first dose, sixty centigrammes more may be given, which generally determines a prompt separation of the placenta, and its expulsion through the os.

Sometimes, unfortunately, the ergot occasions a tetanic constriction of the neck, and complete imprisonment of the placenta. This accident, however, rarely occurs during the first half of gestation. On the other hand, a uterus exhausted by long continued labor, is peculiarly liable to ergotic tetanus. The accident must be combated by baths, emollients, and narcotics. Since ergot alone does not prevent hemorrhage, a tampon must always be at hand, to provide against this latter exigency.

M. Gueniot ranks the foregoing agents in the order in which I have specified them; and considers the use of the finger, or of intra-uterine injections as ultimate resources, to be appealed to only when others fail.

In abortions of the fifth and sixth month, the accidents provoked by the retention of the placenta are nearly as formidable as at term. In this case, the dilatability of the vagina and of the os enables the placenta to be directly extracted by the hand, and this proceeding is indicated as soon as the delay of twelve or six hours has been overpassed.

In the second class referred to above, where accidents exist, these demand attention before everything else. Whether the normal delay be accomplished or not, it is incumbent on the physician to intervene in the artificial extraction of the placenta as soon as abundant hemorrhage or signs of purulent injection are manifested.

If the placenta be only partly engorged in the os, and its principal portion be retained above the internal sphincter, it is better to have recourse to ergot than to traction, which may tear the mass. In case of hemorrhage, ergot should be employed conjointly with the tampon. In case of purulent infection, ergot and intra-uterine injections, with internal tonics.

Ergot should not be employed if the purulent infection dates from several days, as, in that case, the uterus may be considered to be stupefied and paralyzed.

In the third case, the doubt concerning the expulsion of the placenta will be removed if accidents occur, or if, after prolonged waiting and repeated examinations, there is no accident.

In the fourth case, since by the supposition the accidents are supposed to exist *after* the expulsion of the placenta, this treatment becomes a problem of general therapeutics.

In the fifth case, as soon as the miscarriage is recognized to be inevitable, the physician should endeavor to favor its accomplishment.

### SPONGE-PESSARIES.

The distinguished physician, Guendan de Mussy, also contributes a note to the *Bulletin*, on the use of sponge-pessaries, of which he highly approves. He remarks that a reaction has taken place against the exaggerated ideas of physicians some years ago, who regarded all deviations and flexions of the uterus as pathological conditions requiring active medical intervention. But the reaction has carried opinion as much too far the other way, and it is too often ignored, these anomalous positions may seriously derange the circulation, or, in predisposed patients, determine all sorts of sympathetic nervous affections. The pessary comforts the patient, and if it does not cure the disease, at least puts the organ into favorable conditions. Where the uterus is conical, the sponge should be depressed in the middle, like a mushroom, to receive the neck. In cases of retroflexion, the sponge should be spread out in a fan-shaped form, having a central prominence to support the *museau de tanche*, while the limb of the fan sustains the *cul-de-sac.*

It is recommended to coat the lower third of the sponge with yellow-wax, to render it impermeable to the urine. The wax should be melted, the sponge immersed in it, and allowed to cool, and again immersed, and so on until a thick coat be formed.

### TREATMENT OF DYSENTERY.

Dr. Beaufort has presented several considerations on the treatment of dysentery, which disease he considers to be essentially allied, as to its cause, with intermittent fever. Both depend on the combination of heat and damp in the weather. If dampness is occasional, and heat intense and constant, intermittent fever is produced; if the dampness is constant, and the heat liable to brusque variations, dysentery occurs. The common cause is the effluvia from decomposing vegetable matter. One of the first phenomena is congestion of the system of the portal vein, and relief of this congestion constitutes the ultimate therapeutic indication. In the treatment of dysentery, it is necessary, first, to empty the large intestine of altered and irritating mucus, by means of evacuants and absorbing alkalines, (chalk and subnitrate of bismuth;) second, coagulate sanguinolent and albuminoid matters on the surface of the intestine, by means of injections of neutral perchloride of iron, ten to twelve drops in half a lavement of warm water, repeated two or three times in the twenty-four hours. By this means the mucous membrane becomes protected and enabled to heal. Third, diminish congestions of the portal system, sometimes by leeches at the anus, but principally by the use of ipecacuanha, which acts as a stimulant to the vaso-motor system.

### GANGRENE OF THE MOUTH.

Dr. Leavit reports a case of gangrene of the mouth occurring in a child of three years old, already affected with tuberculous enteritis, and which he succeeded in curing by the application of creosote in which camphor had been dissolved. The disease commenced with two ulcerations, round, hollow, white, which were situated in the gingivo-labial fold. Their contents were characteristic, grumulous, dry, and white. In twenty-four hours the ulcerations had greatly extended, were black at the periphery, grumulous at the centre, the mucous fold toward the nose was profoundly excavated, the gum superficially destroyed in an extent of fifteen millimetres, and a fistulous point existed under the lip. An odor of gangrene was exhaled. On this day, for the first time, three cauterizations were made with the camphorated creosote. The next day notable amelioration, inflammatory reaction. In the evening, cauterization with camphorated creosote diluted with alcohol. Two more powerful cauterizations and several with the diluted fluid were made subsequently, and ten days after the first, the cure was complete.

### CROUP CURED BY TRACHEOTOMY.

At the Societé des Hopitaux on the séance of the 8th, M. Roger mentioned a case of croup cured by tracheotomy. Several days elapsed before the canula could be withdrawn, for the child immediately exhibited symptoms of suffocation as soon as the attempt was made. Recourse was at last had to Faradisation of the larynx, and from the day of the first application of electricity, the little patient was able to breathe without the canula during the day, if only it was replaced at night. The tube was not definitely withdrawn till a month after the operation.

At Hotel Dieu, M. Moissenet has had a case of membranous angina confined to the right tonsil. This organ was covered with a thick layer of membrane, having a fetid odor. The disease was overcome by applications of tincture of iodine.

M. Moissenet considers diphtheria less dangerous when it attacks the tonsil, than when it invades the soft palate.

Also at Hotel Dieu, M. Isambert, in speaking of a slight epidemic of small-pox that had occurred in his wards, took occasion to praise highly the use of repeated warm baths, which he did not fear to commence even during the period of the eruption. The warm bath, (given of course in the ward,) has the advantage of diminishing the burning heat of the skin, suppressing the inflammatory areola which surrounds the base of the papulæ, without hindering their suppuration. The cicatrization seems to be less deep and indelible. Emunction with mercurial ointment, was also found to be very useful in preventing cicatrices on the face, without occasioning the painful constriction determined by collodion.

At the Hospice des Incurables, recently died a patient affected with leurocythemia. At the autopsy, the spleen presented the following diameters. Vertical 32 centimetres, transversal 22, antero-posterior 19. It weighed two kilogrammes, its tissue was softened, and contained an infarctus. The liver also was hypertrophied. The kidneys the seat of fatty degeneration, although no albumen had been discovered in the urine. The heart was fatty, and the large vessels filled with clots. Some granulations existed on the mucous surface of the large intestine, and two infarctus in the duodenum.

M. Dumontpallier communicated a case of severe hiccup, which was finally cured by electricity. One pole of the battery was placed on the cervical vertebræ near the origin of the phrenic nerve, the other on the border of the cartilage of the last ribs towards its sternal extremity. Hardly had the current traversed the chest, when the patient uttered a loud cry, the hiccup became transformed into a sob, and ceased abruptly. It reappeared two hours afterwards, and yielded again to electricity, complete interval of nine days, then the hiccup reappeared during several hours, but this time was definitely cured by electrization.—*Med. and Surg. Reporter.*

